# The Immediate Effects of a Combined Mass Drug Administration and Indoor Residual Spraying Campaign to Accelerate Progress Toward Malaria Elimination in Grande-Anse, Haiti

**DOI:** 10.1093/infdis/jiab259

**Published:** 2021-05-16

**Authors:** Thomas Druetz, Gillian Stresman, Ruth A Ashton, Vena Joseph, Lotus van den Hoogen, Matt Worges, Karen E S Hamre, Carl Fayette, Frank Monestime, Daniel Impoinvil, Eric Rogier, Michelle A Chang, Jean Frantz Lemoine, Chris Drakeley, Thomas P Eisele

**Affiliations:** 1 Center for Applied Malaria Research and Evaluation, School of Public Health and Tropical Medicine, Tulane University, New Orleans, Louisiana, USA; 2 Department of Social and Preventive Medicine, School of Public Health, University of Montreal, Montreal, Quebec, Canada; 3 Centre de Recherche en Santé Publique, Montreal, Quebec, Canada; 4 Department of Infection Biology, London School of Hygiene and Tropical Medicine, London, United Kingdom; 5 Malaria Branch, Division of Parasitic Diseases and Malaria, Center for Global Health, Centers for Disease Control and Prevention, Atlanta, Georgia, USA; 6 CDC Foundation, Atlanta, Georgia, USA; 7 IMA World Health, Port-au-Prince, Haiti; 8 Programme National de Contrôle de la Malaria, Ministère de la Santé Publique et de la Population, Port-au-Prince, Haiti

**Keywords:** malaria, mass drug administration, indoor residual spraying, *P. falciparum*, Haiti, ecological study, elimination strategies

## Abstract

**Background:**

Haiti is planning targeted interventions to accelerate progress toward malaria elimination. In the most affected department (Grande-Anse), a combined mass drug administration (MDA) and indoor residual spraying (IRS) campaign was launched in October 2018. This study assessed the intervention’s effectiveness in reducing *Plasmodium falciparum* prevalence.

**Methods:**

An ecological quasi-experimental study was designed, using a pretest and posttest with a nonrandomized control group. Surveys were conducted in November 2017 in a panel of easy access groups (25 schools and 16 clinics) and were repeated 2–6 weeks after the campaign, in November 2018. Single-dose sulfadoxine-pyrimethamine and primaquine was used for MDA, and pirimiphos-methyl as insecticide for IRS.

**Results:**

A total of 10 006 participants were recruited. Fifty-two percent of the population in the intervention area reported having received MDA. Prevalence diminished between 2017 and 2018 in both areas, but the reduction was significantly larger in the intervention area (ratio of adjusted risk ratios, 0.32 [95% confidence interval, .104–.998]).

**Conclusions:**

Despite a moderate coverage, the campaign was effective in reducing *P. falciparum* prevalence immediately after 1 round. Targeted MDA plus IRS is useful in preelimination settings to rapidly decrease the parasite reservoir, an encouraging step to accelerate progress toward malaria elimination.

## Teaser

A mass drug administration campaign against malaria (combined with indoor residual spraying) was carried out in 2018 in Grande-Anse, Haiti. The campaign was significantly associated with an immediate 68% reduction in *Plasmodium falciparum* prevalence.

Haiti is 1 of the only 2 Caribbean countries with endemic malaria transmission. Most (>99%) infections are due to *Plasmodium falciparum*, with only sporadic reports of *Plasmodium vivax* and *Plasmodium malariae* [1, 2] and *Anopheles albimanus*, the main vector [3–5]. The country is committed to eliminating malaria, thanks to a favorable context including parasite prevalence detected by polymerase chain reaction (PCR), consistently estimated at <1% in national surveys [1, 6–11].

To that end, the National Malaria Control Programme in Haiti has implemented a number of interventions over the last decade. Systemwide changes were introduced, such as the introduction of rapid diagnostic tests (RDTs), the addition of primaquine (PQ; 0.75 mg/kg in a single dose) to chloroquine (25 mg/kg administered over 3 days) as first-line treatment, the strengthening of surveillance and laboratory capacities, and a nationwide distribution of long-lasting insecticidal nets (LLINs) in 2012, with a top-up distribution in high-transmission areas in mid-2017 [12–14].

Targeted interventions have also been introduced as malaria transmission is highly heterogeneous in the country [15–17]. In 2015, the Malaria Zero Consortium (https://www.malariazeroalliance.org/) was created to support the acceleration toward elimination and provide formative evidence that will assist in tailoring strategies [12, 18–20]. Mass treatment campaigns were considered, since studies conducted in low-endemic settings have shown their feasibility, effectiveness in reducing malaria prevalence, and potential contribution to shorten the timeline to elimination if combined with other interventions [21–24]. Mass drug administration (MDA) is well suited to elimination settings because of the asymptomatic reservoir; the high proportion of low-density infections makes detection and targeting challenging [25]. However, models indicate that the positive effects of MDA in low-transmission settings are temporary [26]. Therefore, the World Health Organization recommends MDA in areas approaching interruption of transmission, with limited risk of reimportation, and after scale-up of other interventions [27, 28].

As recommended, targeted MDA (tMDA) was only considered in Haiti once the passive surveillance system was strengthened, and after the introduction of community case management and removal of user fees in health facilities [28]. Aiming to rapidly reduce malaria transmission in the most afflicted department, a tMDA campaign using sulfadoxine-pyrimethamine (SP) and single low-dose primaquine (SLD-PQ) was implemented in a single round. It was implemented on top of a vector control strategy that included prior population-wide distribution of LLINs and targeted indoor residual spraying (tIRS) using the insecticide pirimiphos-methyl [29]. The campaign targeted the entire population residing in the areas of highest malaria transmission. There is some evidence that MDA campaigns are acceptable and feasible in Haiti [12, 30]. However, this is the first time in decades that a malaria MDA has actually been used in Haiti. We used an ecological quasi-experimental study design (pre–post with nonrandomized control group) to evaluate the immediate effects of this targeted campaign on malaria prevalence.

## MATERIALS AND METHODS

### tMDA + IRS Campaign

**Figure 1.**
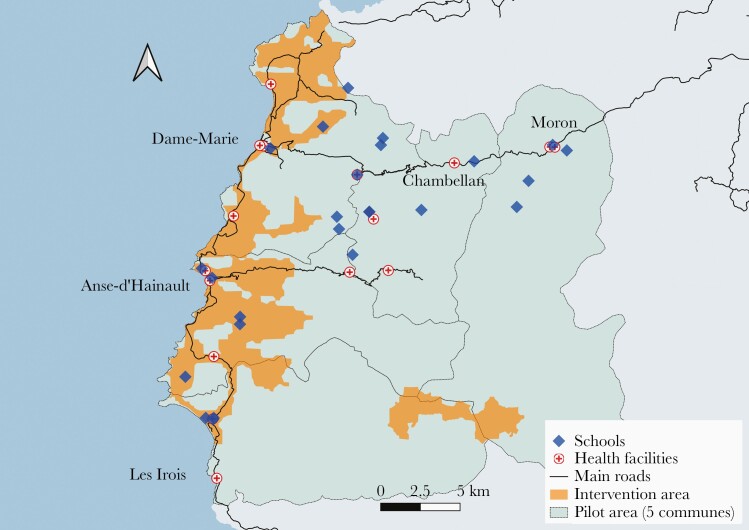
Map of the 5 communes of the pilot area in Grande-Anse Department, Haiti. The 41 easy access groups are represented as diamonds (for schools) or as crosses (for health facilities). The intervention area targeted for mass drug administration and indoor residual spraying is displayed in orange (or grey in printed version).

The intervention campaign took place 10 October–6 November 2018, in 5 communes of Grande-Anse Department, just before the annual seasonal peak of malaria. This department has the highest malaria incidence rate in the country (18.1 per 1000 in 2017, compared to 1.7 per 1000 nationally). The pilot area comprised 5 communes selected based on epidemiological, spatial, logistical, and social factors (Figure 1). Within these communes, the intervention area was restricted to 12 operational units, defined as the contiguous polygonal areas of approximately 2000 residents with the highest predicted reproductive numbers. Models that integrated population density, surveillance data, population mobility scores, and ecological factors were used to predict risk of transmission and rank operational units. As potential sources of malaria transmission to the whole area, targeting the units with the highest current reproductive number would likely have spillover effects and reduce overall risk of infection [31].

Following a census, every household was visited and offered a treatment that comprised a single dose of SP + SLD-PQ (SP-PQ). The target dose for SP was 25/1.25 mg/kg, the approved therapeutic dose in Haiti for second-line treatment. The target dose for PQ was 0.25 mg/kg, lower than the recommended therapeutic dose. SP and SLD-PQ were chosen because they can be administered in a single dose and they have different therapeutic effects [32]. There is no indication of widespread *P. falciparum* resistance to PQ or SP in Haiti [8].

All individuals aged ≥6 months were offered directly observed, age-appropriate treatment of SP-PQ in a single dose. Women in their first trimester of pregnancy and participants with signs of severe illness, known allergies to SP or PQ, specific medical conditions, or using contraindicated medications were excluded. Pregnant women in their second or third trimester and breastfeeding women were offered SP only. Return visits or mop-up distribution were arranged for those temporarily absent [29].

Simultaneously, a separate team led a tIRS campaign in the same area. The organophosphate insecticide pirimiphos-methyl (Actellic 300CS) was applied once to each dwelling. It has a long residual activity (5–9 months) and no reported resistance in Haiti. Spraying was conducted after all individuals, animals, and large pieces of furniture were removed from the household. Wall bioassays were performed to confirm quality of the insecticide application.

### Study Design

This is an ecological, quasi-experimental study, using a pre- and posttest with nonrandomized control group to assess the effectiveness of tMDA + IRS on malaria parasite prevalence at the venue level [33]. Surveys were conducted 6 November–7 December 2017 and 12 November–13 December 2018 in a panel of easy access groups (EAGs). Participants were recruited among the persons attending the EAG sites at the time of the survey. With a short lapse of time (1–5 weeks) between the campaign and the 2018 survey, the present study is designed to assess the intervention’s immediate effects.

The intervention group includes all participants recruited in the EAGs located in intervention area. The control group includes the participants recruited in EAGs located in nontargeted areas. Exposure is determined based on the EAG location, not on individual self-reported exposure to the intervention, nor on household location. More information about the EAG surveys is available elsewhere [16]. The Strengthening the Reporting of Observational Studies in Epidemiology (STROBE) guidelines were followed (Supplementary File 1).

### Pilot Area and EAG Sampling

The pilot area has a total estimated population of 156 138 within an area of 582 km^2^ (Figure 1). It is located in southwest Haiti, an approximately 10-hour drive from the capital. The pilot area is characterized by diverse environmental conditions: high mountains, rivers, lowlands, valleys, and dense forests. Three of the 5 included communes share a contiguous coastline. The population is mostly rural and hard to reach, although a few towns have ˂20 000 inhabitants. Within the 5 communes, the area targeted for MDA + IRS covers 98 km^2^, with an estimated population of 46 372.

Two types of EAGs were sampled in the pilot area: primary schools and health facilities (Figure 1). All health facilities (n = 16) in the pilot area were included. For schools, after a census of all primary schools with at least 100 pupils, stratified random sampling was used to select 25 schools and ensure equal distribution across communes and by remoteness. The same EAGs were surveyed in both years of the study. More information is available elsewhere [16].

### Participants and Survey Procedures

All new attending and accompanying persons in the health facilities were eligible to participate, except those who were attending a scheduled visit or required urgent care. In schools, all pupils were enrolled if their total number per school was <150; otherwise, a simple random sample of 150 children was selected. A total of 5000 participants were surveyed at each survey round. Participants were categorized into the intervention or control group based on the location of the EAG where they were recruited.

A sociodemographic questionnaire was administered to all participants. A capillary blood sample from a finger-prick was taken to perform a conventional histidine-rich protein 2 (HRP2)–based RDT (SD Bioline Ag. Pf, South Korea). If invalid, it was repeated. Finger-prick blood was also spotted on Whatman 903 cards (GE Healthcare), dried overnight at ambient temperature, and packed the next day with silica gel. The detailed procedure for recruiting and replacing participants is described elsewhere [16]. Refusal and dropout rates were <1%.

For the participants with RDT-positive results, confirmation of *P. falciparum* infection was obtained by PCR [34]. Individuals with a positive RDT were provided the recommended first-line treatment. All participants testing positive by RDT and a random selection of 30% of those testing negative were traced to their household, where spatial coordinates were recorded using GPS devices (Garmin, Olathe, Kansas).

### Outcome and Statistical Analyses

The unit of analyses is the EAG. The outcome for this study is the *P. falciparum* prevalence, estimated by the proportion of participants with a PCR-confirmed positive HRP2-based RDT. Because of the pseudo-panel structure of the study (EAGs being time-invariant, not the participants), effects could not be evaluated at the individual level. Data were therefore aggregated using the cross-groups averaging method [35, 36]. Intention-to-treat analysis was used; individuals within EAGs that were targeted for MDA + IRS were considered exposed (intervention group), whereas participants sampled from EAGs outside the targeted area were considered unexposed (control group).

The average treatment effects were expressed as the ratio of adjusted risk ratios (RaRRs); that is, the relative pre–post change in prevalence was compared between the intervention and control groups. This approach enables controlling for observed and unobserved time-invariant confounders [37, 38]. RaRR (a relative term) is more appropriate than difference-in-differences (an absolute term) to assess changes when the 2 baseline measures differ [39].

Due to overdispersion, a negative binomial regression model was fitted with the total count number of positive HRP2-based RDTs as the dependent variable, and the number of RDTs performed as the offset [40]. Potential time-varying confounding variables were tested in the model: sociodemographic characteristics, use of LLINs, travel history, and total rainfall during the previous 2 months. The final model included LLIN use (averaged at the venue level) and rainfall (at 5 km resolution), with the best-fitting model selected according to the Akaike information criterion values. Cluster-robust variance estimators were consistently used [41].

Sensitivity analyses were conducted by splitting the intervention group into 2 subgroups, with the median MDA coverage among the EAGs located in the intervention area (60%) used as the cutoff. The exposure variable was therefore redefined into 3 categories: control group, low MDA coverage (<60%), and high MDA coverage (≥60%).

All analyses were performed using Stata version 14.0 software (StataCorp LLC, College Station, Texas). Maps were produced using QGIS version 3.8.1 Zanzibar (open-source software with general public license). Rainfall data were extracted from the climate hazards precipitation with station database.

### Ethical Considerations

Consent procedures are detailed elsewhere [16]. In health facilities, informed written consent was sought from adult participants and from parents/guardians of children (<18 years of age). In schools, an opt-out method was used to obtain consent from the children’s parents. Written assent was sought for children >6 years of age. Participants could choose to give thumbprint consent/assent if they could not sign.

The study was approved by the National Bioethics Committee in Haiti (1516–30), the London School of Hygiene and Tropical Medicine Ethics Committee (103939), and the Tulane University Institutional Review Board (795709). Participation in the study was not remunerated. Activity did not constitute engagement in human subjects research as determined by the US Centers for Disease Control and Prevention’s Center for Global Health human subjects office (number 2016-135a).

## RESULTS

### Study Participants

A total of 10 006 participants were recruited in 41 EAGs (Table 1), 19 of which were located in the intervention area (Figure 1). In 2017, 48% of the 5026 participants were recruited in the area that was later targeted for intervention. In 2018, 42% (2094 of 4980) of the participants were recruited in the intervention area, of which 59% (n = 1238) reported that their household was visited for the campaign. Among these, 86% (n = 1089) reported having taken MDA in the previous weeks. The primary reasons for not having taken MDA despite the household visit were being absent (n = 74), being excluded (n = 37), and refusal (n = 12). Among the participants recruited in 2018 in the control area, <2% (n = 47) reported having been exposed to MDA. Regarding IRS, 33% (n = 683) of participants recruited in the intervention area reported that their household had been sprayed in the previous weeks vs 6% (n = 168) in the control area. In the intervention area, PCR-confirmed RDT positivity was significantly associated in bivariate analysis with self-reported exposure to MDA (odds ratio [OR], 0.15 [95% confidence interval {CI}, .070–.356]), but not to IRS (OR, 0.73 [95% CI, .374–1.430]). About 86% (2017) and 92% (2018) of RDT-positive samples were confirmed by PCR.

**Table 1.** Participant Characteristics, by Intervention Area and Year

	Intervention Area			Control Area		
Characteristic	2017	2018	*P* Value	2017	2018	*P* Value
Participants, No.	2425	2094		2601	2886	
Female sex	0.56	0.59	.092	0.51	0.55	.003
Slept under a bed net the night before	0.57	0.47	<.001	0.42	0.27	<.001
Age group, y						
<5	0.11	0.15	.001	0.1	0.13	<.001
5–14	0.46	0.42		0.49	0.51	
15–29	0.25	0.24		0.23	0.22	
30–45	0.09	0.1		0.09	0.07	
>45	0.09	0.09		0.1	0.07	
Traveled in the past 3 mo	0.03	0.04	.004	0.04	0.03	.119
History of fever in the past 2 wk	0.16	0.15	.692	0.11	0.13	.022
Household size >5	0.52	0.53	.207	0.55	0.58	.049
Household owns livestock	0.45	0.53	<.001	0.61	0.63	.165
Household owns bed net(s)	0.68	0.59	<.001	0.57	0.39	<.001
Occupation of the head of the household						
Farmer	0.54	0.41	<.001	0.71	0.62	<.001
Shopkeeper	0.25	0.37		0.14	0.21	
Other	0.21	0.22		0.15	0.17	
Urban area	0.59	0.6	.422	0.28	0.28	.695
Commune						
Moron	0.27	0.29	.304	0	0	<.001
Chambellan	0.36	0.34		0	0	
Dame-Marie	0.24	0.25		0.36	0.37	
Anse-d’Hainault	0.07	0.06		0.48	0.42	
Les Irois	0.06	0.06		0.16	0.21	
Took MDA	0	0.54	<.001	0	0.02	<.001
Household was sprayed (IRS)	0	0.34	<.001	0	0.06	<.001

Abbreviations: IRS, indoor residual spraying; MDA, mass drug administration.

Data are presented as proportions in each group.

### Effects on *P. falciparum* Prevalence

**Figure 2.**
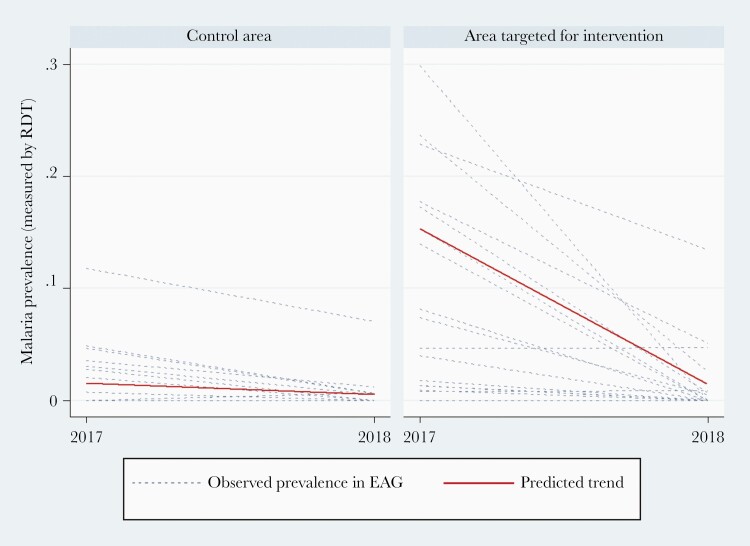
Observed and predicted trends in *Plasmodium falciparum* prevalence per easy access group (EAG) between 2017 and 2018. Parasite prevalence was measured by rapid diagnostic test (RDT), with polymerase chain reaction confirmation of positive cases. Predicted trend was derived from a negative binomial model with the total number of positive cases as the dependent variable, the total number of tests performed as the offset, and the area type (intervention vs control) as exposure. The model was adjusted for potential time-varying confounding variables.

At baseline, prevalence was higher in the EAGs located in the intervention area (ranging from 0 to 30%) compared to the control area (ranging from 0 to 12%) (Table 2). This was expected since the intervention was implemented in the highest-transmission areas. Prevalence diminished between 2017 and 2018 in both areas (Figure 2), but the reduction was significantly larger in the intervention area. Intent-to-treat analysis predicts a 68% further reduction in malaria prevalence (RaRR, 0.32 [95% CI, .103–.998]) after adjusting for LLIN use and rainfall (Table 3).

**Table 2.** Early Access Group (Cluster Sampling Units) Characteristics, by Year and Exposure Area

	Control Area			Intervention Area		
Characteristic	2017	2018	Difference in Means^a^	2017	2018	Difference in Means^a^
Total No. of sites	22	22		19	19	
No. of participants per site	118	130	–12.5	128	110	18.6
Age of participants, y	16	16	–0.8	17	16	–0.7
% of female participants	0.494	0.539	–0.044	0.548	0.571	-0.022
% of participants who slept under a bed net the night before	0.416	0.266	–0.150**	0.546	0.479	–0.068
% of participants who traveled recently	0.027	0.026	–0.001	0.021	0.031	0.010
% of large households (>5 members)	0.552	0.563	0.020	0.532	0.567	0.035
% of households that own cattle	0.624	0.642	0.017	0.458	0.548	0.090
% of farming households	0.741	0.637	–0.104	0.592	0.492	–0.100
Total rain precipitation, mm, over the previous 2 mo	330	230	–100***	330	234	–95***
% of participants with positive RDT (confirmed by PCR)	0.015	0.005	–0.010	0.091	0.017	–0.074**

Abbreviations: PCR, polymerase chain reaction; RDT, rapid diagnostic test.

^a^Tests on the equality of means that are statistically significant at a threshold of 0.05 are marked (**P < .01; ***P < .001).

**Table 3.** Reduction in Malaria Prevalence Following the Targeted Intervention Campaign

Area	Preintervention	Postintervention	Adjusted RR	(95% CI)	*P* Value
Control area	1.32%	0.52%	0.394	(.073–2.109)	.276
Intervention area	14.28%	1.80%	0.126	(.022–.724)	.020
Ratio of adjusted RRs			0.321	(.104–.998)	.049

Abbreviations: CI, confidence interval; RR, risk ratio.

**Figure 3.**
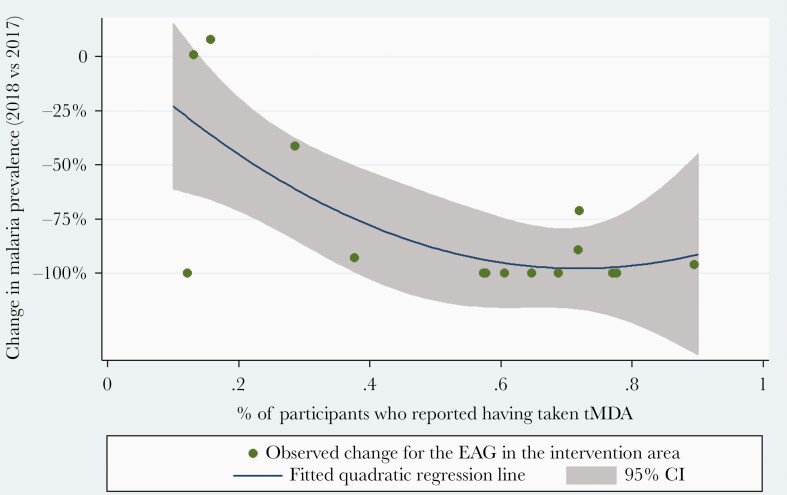
Relative difference in *Plasmodium falciparum* prevalence per easy access group (EAG) between 2017 and 2018, characterized by mass drug administration (MDA) coverage per EAG in 2018. MDA coverage per EAG is defined as the percentage of participants who self-reported having received MDA treatment in the previous weeks. *P. falciparum* prevalence is expressed as the percentage of positive rapid diagnostic tests out of the total number of tests performed per EAG. The association was assessed by fitting a quadratic function (y = α + βx + γx^2^). The coefficient of determination (ie, % of variance explained by MDA coverage) equals 51.17%. Abbreviations: CI, confidence interval; EAG, easy access group; tMDA, targeted mass drug administration.

**Figure 4.**
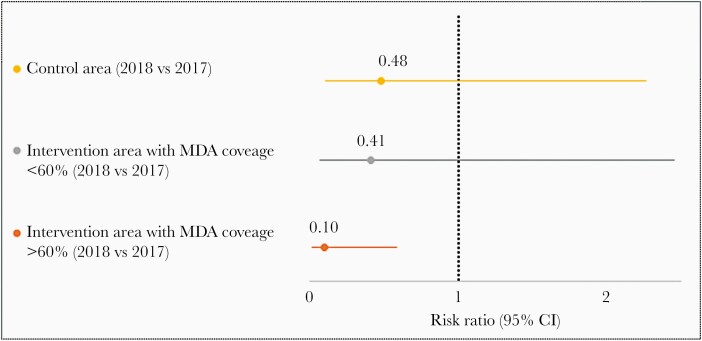
Risk ratios of malaria, 2018 vs 2017, with 95% confidence intervals (CIs) and by level of mass drug administration (MDA) coverage. MDA coverage per easy access group is defined based on the proportion of participants who self-reported having taken MDA in the previous weeks. The exposure variable was redefined based on 3 categories: control area, intervention area with MDA coverage <60%, and intervention area with MDA coverage ≥60%.

Visual observation of the data and the unadjusted coefficient of determination suggested that prevalence was correlated to self-reported exposure to MDA (Figure 3), but not to self-reported exposure to IRS (Supplementary File 2). Sensitivity analyses were therefore conducted by categorizing EAGs in the intervention area as low (<60%) vs high (≥60%) rates of self-reported exposure to MDA (Figure 4). A dose-response gradient was observed. Indeed, when compared to the control area, the EAG with low MDA coverage presents a nonsignificant 15% further reduction in malaria prevalence (RaRR, 0.85 [95% CI, .270–2.719]), in contrast to the 79% reduction in the EAG with high MDA coverage (RaRR, 0.21 [95% CI, .054–.812]).

### Sensitivity Analysis

A per-protocol analysis was also performed for “adherent” participants (n = 9017)—those who either reported having taken MDA and were recruited in the intervention area, or reported having not taken MDA and were recruited in the control area. Per-protocol analysis suggests that the intervention is associated with an 86% decrease in *P. falciparum* prevalence (RaRR, 0.14 [95% CI, .037–.573]).

## DISCUSSION

The findings indicate that the MDA + IRS campaign was associated with an immediate reduction in malaria parasite prevalence by 68%, which was statistically significant but with a wide uncertainty range. Sensitivity analyses suggest that the effectiveness would have increased if more of the participants recruited in the intervention area would have been exposed to it. In the subgroup of EAGs with tMDA coverage ≥60%, its effectiveness in reducing malaria prevalence reached 79%, close to the 86% effectiveness obtained in the per-protocol analysis.

Our estimate is smaller than the 97% reduction within 1 month post-MDA that was found in a recent meta-analysis of studies conducted in settings with moderate endemicity [21], but those studies took place decades ago in Kenya and India and did not use the same drug regimen. Results from the present study are also difficult to compare with the evaluation of repeated MDA campaigns that took place in Haiti in the 1960s using chloroquine and pyrimethamine [42]. The true effect is likely underestimated in the present evaluation, most importantly because of the moderate MDA (54%) and IRS (33%) coverage in the intervention area. Misclassification errors between the targeted and control areas are also possible since catchment populations of EAGs do not perfectly overlap with these respective areas, although this error concerned only 2.7% (46/1682) of the geolocated households (Supplementary Files 3 and 4). These factors would bias the results toward a null effect.

This study cannot disentangle the effects of the 2 components of the intervention, since they overlapped in time and space. However, it is unlikely that the estimated effects can be attributed to the tIRS component. Indeed, IRS generally requires intensive campaigns to reduce malaria transmission, with high coverage (>80%) and multiple rounds of administration [43, 44]. In addition, they do not affect the parasite reservoir in infected individuals, but rather reduce transmission and protect the population from later resurgence [45]. Unsurprisingly, our analyses have not found evidence of an association between self-reported exposure to IRS and malaria prevalence in the targeted sites.

Under such conditions, the estimated prevalence reduction is very encouraging, especially after only 1 round. Targeting the areas with the greatest risk of malaria transmission immediately reduced the gap between the low-risk and high-risk zones. When comparing baseline to endline, the proportion of EAGs with prevalence <1% increased from 21% to 68% in the targeted area, and from 68% to 91% in the control area. The impact could be optimized by adding tMDA + IRS rounds and by reaching more people in the intervention area [21, 22]. As prevalence decreases (<3%), the strategy might be refined to identify the remaining asymptomatic reservoir populations and redirect aggressive MDA + IRS campaigns toward them to further progress to elimination [23]. However, the timing of switching strategies is problematic. Indeed, the benefits of MDA are transient, but identifying the asymptomatic reservoir takes time and remains difficult until overall transmission is already low [25, 46].

Targeted MDA + IRS is recommended when foci are clustered in small areas, especially with high population mobility [23]. Studies conducted in Grande-Anse have suggested that malaria infections are locally acquired; the department was portrayed as a source rather than a sink of cases [11, 16]. By targeting the areas with the highest predicted malaria risk in Grande-Anse, the intervention was expected to benefit the individuals not only in—but also outside—these areas, whether they took MDA (or received IRS) or not. Our results are congruent with (but cannot establish) the presence of a “community effect,” since malaria prevalence also decreased in the control area and among those nonexposed in the intervention area, even after adjusting for rainfall.

This is an ecological study and, as such, does not purport to assess causal inference or draw conclusions at the individual level. Rather, it examines the intervention’s effects on malaria prevalence in the overall catchment population of EAG venues. The aggregation of individual data at the EAG level was required to obtain a panel structure and strengthen the robustness of the evaluation design. Unfortunately, this rendered the study ecological and reduced statistical power of the analysis. Furthermore, the surveys in the EAGs were planned before, and independently of, the intervention. Both surveys were carried out during the same period of the year to increase their comparability, even if it meant examining only the immediate effects of the intervention.

Only a portion of the EAG catchment populations (ie, those who live in the areas with the highest predicted risk) were targeted for MDA + IRS. It was therefore anticipated to assess an intervention whose coverage would be moderate at best. More than a limitation, this constitutes one of this study’s unique characteristics. Instead of randomized clinical trial conditions, this is one of the first evaluations of a highly targeted MDA + IRS campaign [47]. This echoes our intention to inform programmatic efforts about potential strategies to accelerate progress toward malaria elimination, rather than to establish the protective effects of MDA campaigns in Haiti using SP-PQ—likely to be exceptionally high due to the absence of resistance. In the same vein, this study does not claim to assess MDA + IRS coverage or *P. falciparum* prevalence in the general population.

Repeated surveys in EAGs are helpful in designing quasi-experimental studies, even if the intervention is not implemented by the research team (natural experiment design) [48]. The difference-in-differences approach that was used allowed to control for time-invariant observable and nonobservable confounding factors. The influence of potential time-varying confounding factors, such as rainfall and LLIN usage, was tested and adjusted for. Analyses, including bilateral tests and cluster-robust variance estimators, were intentionally conservative. However, the disparity in malaria prevalence at baseline between the control and intervention groups may have affected the observed effect size. RaRRs were preferred over difference-in-differences to minimize this risk. It is still possible that trends in relative changes were dissimilar during preintervention period between the 2 groups. Unfortunately, this hypothesis could not be tested due to the lack of prebaseline survey data. Although an imperfect proxy for prevalence, passive surveillance data do not suggest different preintervention trends between the groups of health facilities.

Other interventions were implemented during the time interval, which might have affected our estimates. However, the context was closely monitored, and exposure to other types of interventions (such as LLINs distribution) was controlled for in the models. Finally, information bias is possible, especially in school-aged children. Several measures were taken to minimize this risk (described elsewhere [16]), which, in any case, is unlikely to be different according to RDT status.

## CONCLUSIONS

This study measured the immediate effects associated with a tMDA + IRS campaign against malaria in Grande-Anse Department, Haiti. The campaign was restricted to the areas with the highest predicted malaria risk. While coverage was only moderate in the study population, the campaign was significantly associated with a 68% reduction in malaria prevalence immediately after 1 round. Further evaluation of the campaign is being conducted and will be published in forthcoming papers. Targeted MDA + IRS can be used in preelimination settings to rapidly reduce malaria transmission, which is an encouraging step to accelerate progress toward elimination depending on local vectorial capacity and importation risk. Repeated surveys in easy access groups provide an evaluation framework for programmatic interventions and natural experiments.

## Supplementary Data

Supplementary materials are available at *The Journal of Infectious Diseases* online. Consisting of data provided by the authors to benefit the reader, the posted materials are not copyedited and are the sole responsibility of the authors, so questions or comments should be addressed to the corresponding author.

jiab259_suppl_Supplementary_File_1

jiab259_suppl_Supplementary_File_2

jiab259_suppl_Supplementary_File_3

jiab259_suppl_Supplementary_File_4

## Notes

***Author contributions.*** T. P. E., C. D., T. D., G. S., J. F. L., and M. A. C. conceived the study. T. D., G. S., R. A. A., L. v. d. H., M. W., V. J., K. E. S. H., C. F., and F. M. were involved in data collection. E. R. and C. D. conducted laboratory analyses. T. D. and G. S. analyzed the survey data. T. D. drafted the manuscript. All authors read and approved the final manuscript.

***Acknowledgments.*** We acknowledge the communities in Grande-Anse who enabled this work to take place; IMA World Health for implementing the study; Dr Erold Joseph for his support finding the GPS coordinates of the schools; Sarah Cooper, Alyssa Young, and Brooke Mancuso for acquiring remote sensing data; and the broader Malaria Zero Consortium for its support.

***Data availability.*** All anonymized data and Stata scripts used for this analysis can be made available by contacting the corresponding author under reasonable request.

***Financial support.*** This study was part of the Malaria Zero Consortium (http://malariazeroalliance.org/), funded by the Bill & Melinda Gates Foundation (grant number OPP1114297 to the CDC Foundation). T. D. is a J1 Research Scholar from the Fonds de recherche du Québec–Santé.

***Potential conflicts of interest.*** All authors: No reported conflicts of interest.
