# Supplementary material for: The Immediate Effects of a Combined Mass Drug Administration and Indoor Residual Spraying Campaign to Accelerate Progress Toward Malaria Elimination in Grande-Anse, Haiti
Source: J Infect Dis. 2021 May 16;225(9):1611–20. doi: 10.1093/infdis/jiab259 (PMC9071345; doi:10.1093/infdis/jiab259)
Supplement: jiab259_suppl_Supplementary_File_2 [file jiab259_suppl_supplementary_file_2.docx]

Supplementary File 2


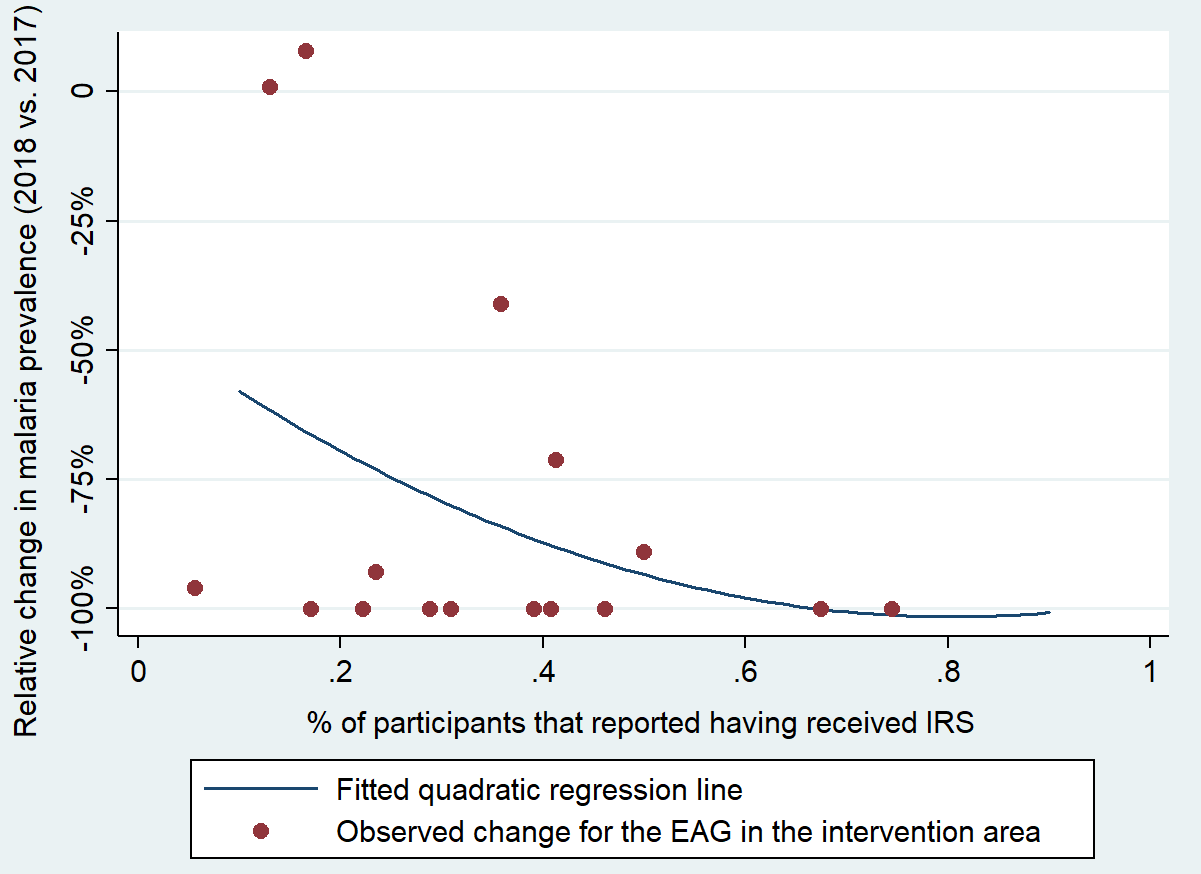


Relative difference in *P. falciparum* prevalence per EAG between 2017 and 2018, characterized by IRS coverage per EAG in 2018. IRS coverage per EAG is defined as the % of participants that self-reported having their house sprayed with insecticide in the previous weeks. *P. falciparum* prevalence is expressed as the % of positive RDT out of the total number of tests performed per EAG. The association was assessed by fitting a quadratic function (y=α+βx+γx^2^). The coefficient of determination (i.e.: % of variance explained by IRS coverage) equals 14.76%. For a better visualization of the figure, the 95% confidence interval around the regression line was not displayed due to its width.

IRS Indoor residual spraying; EAG easy access group; RDT rapid diagnostic test.
