# Supplementary figures and images for: The Immediate Effects of a Combined Mass Drug Administration and Indoor Residual Spraying Campaign to Accelerate Progress Toward Malaria Elimination in Grande-Anse, Haiti
Source: J Infect Dis. 2021 May 16;225(9):1611–20. doi: 10.1093/infdis/jiab259 (PMC9071345; doi:10.1093/infdis/jiab259)

Supplementary File 3


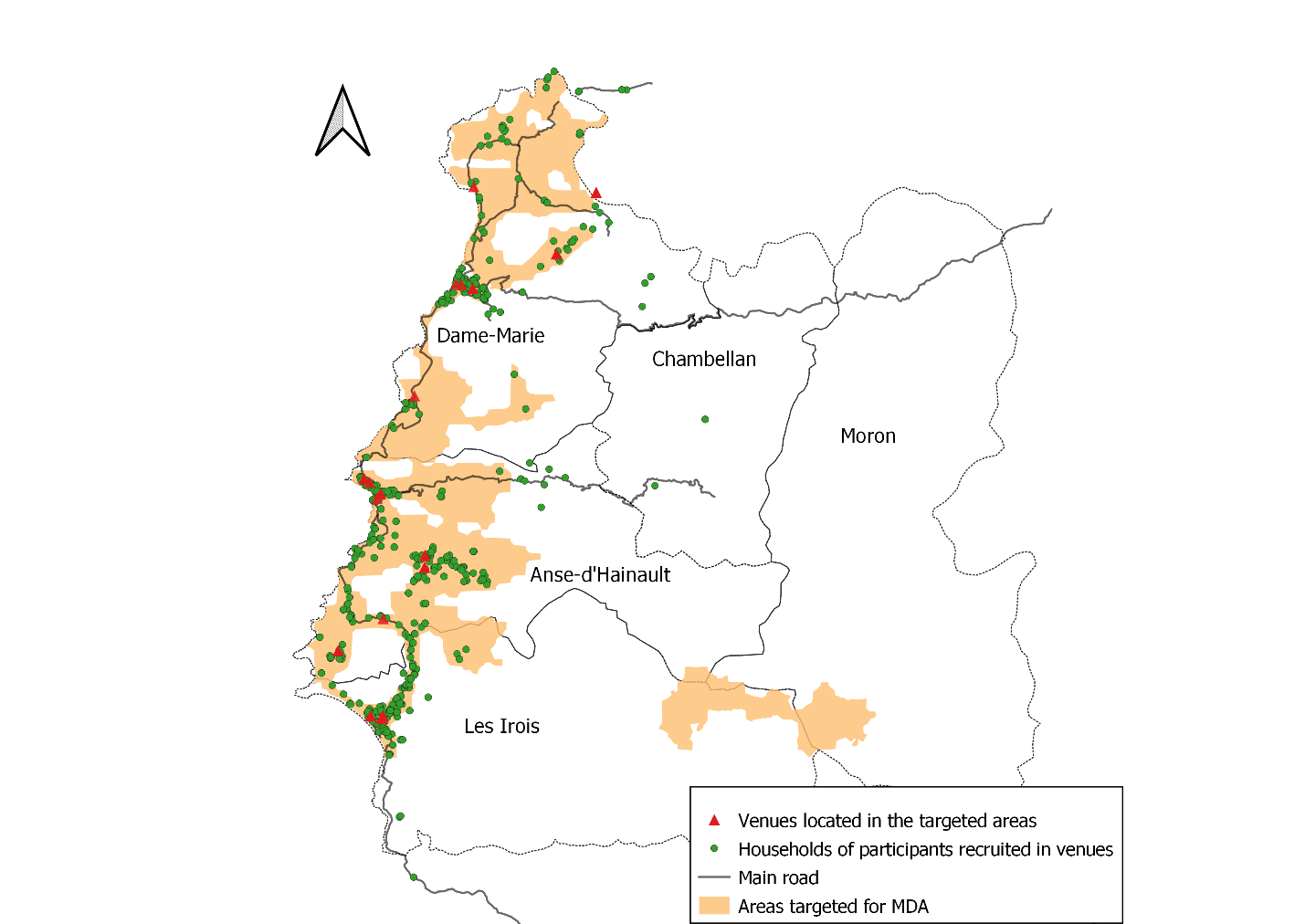

Supplement: jiab259_suppl_Supplementary_File_3 [file jiab259_suppl_supplementary_file_3.docx]

Supplementary File 3


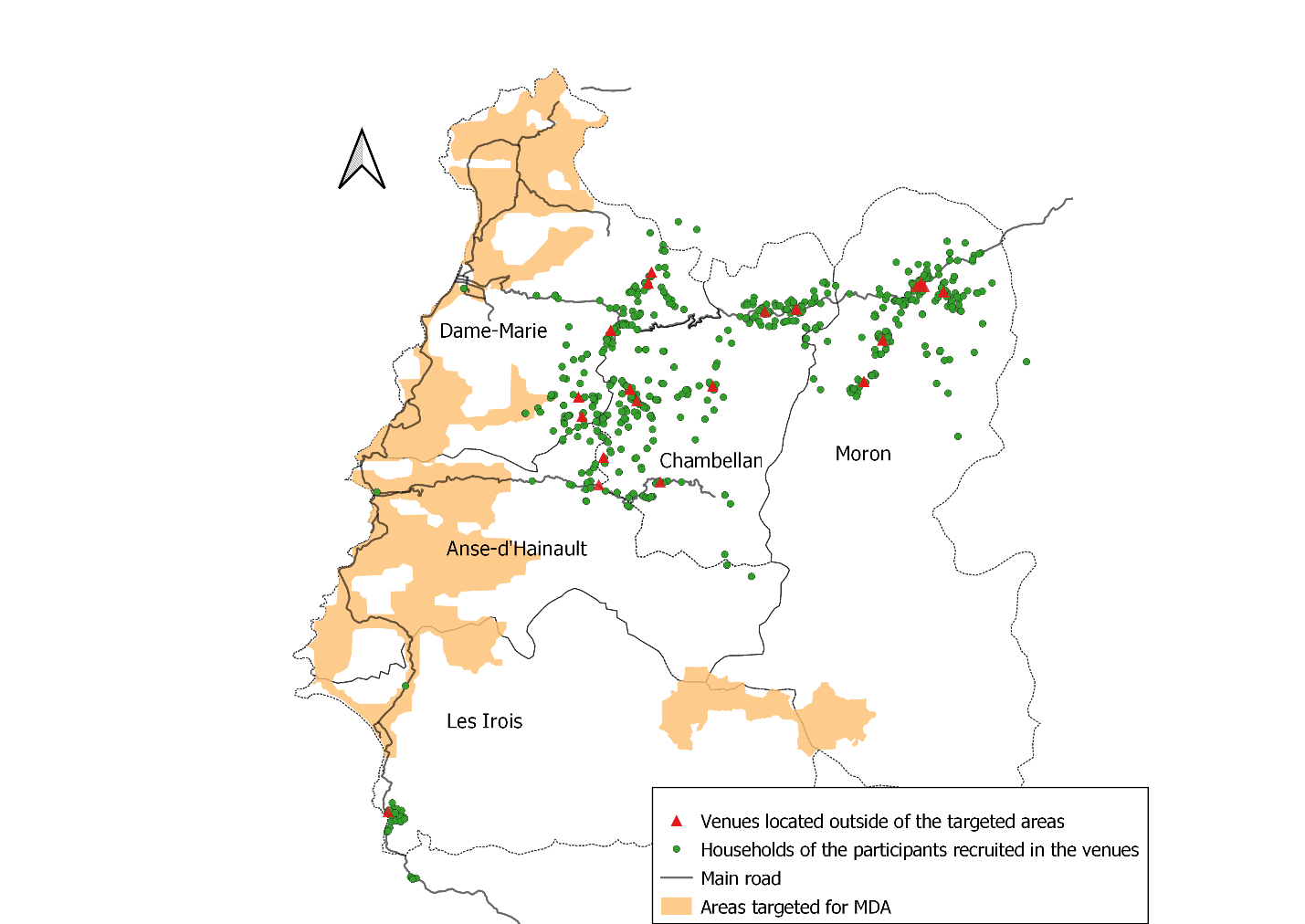

Supplement: jiab259_suppl_Supplementary_File_4 [file jiab259_suppl_supplementary_file_4.docx]
